# A call for open science in forensics

**DOI:** 10.1073/pnas.2321809121

**Published:** 2024-05-23

**Authors:** Thomas D. Albright, Nicholas Scurich

**Affiliations:** ^a^The Salk Institute for Biological Studies, La Jolla, CA 92037; ^b^Department of Psychological Science, University of California, Irvine, CA 92697; ^c^Department of Criminology, Law and Society, University of California, Irvine, CA 92697

**Keywords:** forensic firearms identification, due process, forensic trials, data sharing

## Abstract

The modern canon of open science consists of five “schools of thought” that justify unfettered access to the fruits of scientific research: i) public engagement, ii) democratic right of access, iii) efficiency of knowledge gain, iv) shared technology, and v) better assessment of impact. Here, we introduce a sixth school: due process. Due process under the law includes a right to “discovery” by a defendant of potentially exculpatory evidence held by the prosecution. When such evidence is scientific, due process becomes a Constitutional mandate for open science. To illustrate the significance of this new school, we present a case study from forensics, which centers on a federally funded investigation that reports summary statistics indicating that identification decisions made by forensic firearms examiners are highly accurate. Because of growing concern about validity of forensic methods, the larger scientific community called for public release of the complete analyzable dataset for independent audit and verification. Those in possession of the data opposed release for three years while summary statistics were used by prosecutors to gain admissibility of evidence in criminal trials. Those statistics paint an incomplete picture and hint at flaws in experimental design and analysis. Under the circumstances, withholding the underlying data in a criminal proceeding violates due process. Following the successful open-science model of drug validity testing through “clinical trials,” which place strict requirements on experimental design and timing of data release, we argue for registered and open “forensic trials” to ensure transparency and accountability.

For most of its brief history in the Western world, scientific investigation has been a pastime of an educated elite. Early natural philosophers were effectively skilled craftsmen who traded in unique and prized information. Because information is an easily disseminated commodity, its value in hand promoted a culture of secrecy and selective distribution, which naturally protected the market share of the holders but quashed any potential for the synergy and interdisciplinary development that we cherish in science today.

## The Origins of Open Science

The Scientific Revolution of the late Renaissance saw the emergence of a new economy and a new order (1). The flagbearers of this revolution were often born of industrious families with connections to courts, clergy, arts, or commerce. Their scientific work was primarily supported by a system of noble patronage, fostering an economic model in which royals and lesser aristocrats burnished their reputations and extended their spheres of influence by contracting with the most esteemed scientists of the day. This system promoted significant competition among aspiring patrons, which led to market conditions favorable to a growing scientific enterprise. That enterprise, in turn, began to adopt institutional structures—professional academies and societies—for governance, financial support, and intellectual exchange.

Francis Bacon, 17th-century British philosopher and architect of rational empiricism, idealized this evolution of science toward a utopian community of “Salomon’s House,” which seeks “the knowledge of causes, and secret motions of things; and the enlarging of the bounds of human empire, to the effecting of all things possible” (2). Through a progressive spreading of wings, transparent ascendance, and labor for the collective good, Science accepted Bacon’s challenge to become the rich communal—“open science”—enterprise that exists today.

## The Canon of Open Science

The fundamental rule of open science is that data and tools developed for data acquisition and analysis should be preserved, managed, and made freely available (3). In the most progressive practice, this includes sharing summary-level data, raw data, and the analyzable dataset (e.g., deidentified individual participant data from studies with human subjects), and metadata, or “data about the data” (e.g., protocols, statistical analysis plans, and analytic code) (4).

Responsible sharing of scientific data is not simply a utopian ideal. The reason this rule exists “is that research conducted openly and transparently leads to better science” (3). Historians and social scientists have identified five “schools of thought” (5) that form the canon of open science and highlight practical benefits afforded by broad dissemination of scientific knowledge:

(1) The Public School maintains that by making data accessible and comprehensible, we can engage a larger “citizen science” audience that may play a valuable role in interpretation of results and societal decisions based on science.
(2) The Democratic School states that in a free society with publicly funded efforts to understand the nature of things, everyone must be provided access to the knowledge gained.
(3) The Pragmatic School makes the point that complex and expensive scientific endeavors can be made more efficient by sharing of knowledge and innovation.
(4) The Infrastructure School argues that shared technology, such as distributed computing and core resources, is not simply efficient but also helps to facilitate interdisciplinary science.
(5) The Measurement School is concerned with ways that the real-world impact of scientific discovery can be assessed and improved by new technologies for knowledge sharing.

In short, open access to the fruits of modern science (a) satisfies a contractual obligation to the taxpaying public, (b) increases the efficiency of research and the ability to draw accurate conclusions, (c) allows discoveries to be freely challenged or reinforced through a democratic process, and (d) enables newcomers to rise on the shoulders of those who have gone before.

In the spirit of the many practical benefits of open science highlighted by the five existing schools of thought, we introduce here a sixth school to the open science canon: Due Process. We maintain that sharing of scientific data serves justice in a manner long mandated by the Due Process Clause of the US Constitution. We develop this idea further below after first documenting a recent failure of compliance with open science standards that has important criminal justice implications.

## Resistance and the Modern Evolution of Open Science

Over the years, free and open science has battled numerous contests from capitalist market forces, particularly those stemming from traditional academic economies of promotion, awards, and intellectual property rights; from proprietary control of scientific products; and from the stranglehold that academic publishing has placed on sharing of scientific discovery (6, 7). Driven by mandates from funding agencies, regulatory agencies (8), and professional organizations (e.g., refs. 9–11), new structures and incentives have evolved to combat these forces, such as open-access journals (12), public data repositories (13, 14), and a reconsideration of traditional criteria for professional advancement (15)—all mindful of the fact that both the scientific enterprise, and the people it serves, stand to gain from unbridled cooperation.

Another destabilizing influence is government, which despite its professed role in promoting access to publicly funded knowledge for common benefit (16), now and then takes the path of obfuscation or outright denial of access. One might argue, and many have (e.g., ref. 17), that this path is justified because scientific knowledge offers undue power to bad actors (18). By this argument, scientific secrecy benefits the commodities of security and defense, which are generally valued by sovereign states. In other cases, government prohibition of access to details of publicly funded science is capricious and unfair and surely not in the public interest (19).

## Dark Ages Redux: A Case Study from Forensics

A noteworthy instance of governmental interference is the recent refusal by the Department of Justice to release data from publicly funded research on the validity of forensic methods for criminal investigation. Others have noted that “forensic science” is out of step with mainstream open science practices and have argued for a cultural sea change in this field (20, 21). Here, we present a case study from the forensic discipline of firearms identification, which illustrates the practical consequences of government’s refusal to share data. This refusal is today, in the real world, compromising due process in criminal procedure. We also note that this refusal is not simply in conflict with open science standards and practices but in apparent violation of White House policy on sharing scientific data (16, 22). Drawing from well-established practices for transparent validation of clinical tools in medicine, we show a path to better science and judicial fairness.

## Forensic Firearms Identification

Forensic firearms identification is a species of forensic pattern comparison methods, in which trained observers visually examine patterned impressions of unknown source found at a crime scene and compare them to patterns of known source. Striations and indentations found on a spent bullet cartridge case recovered from a crime scene, for example, are compared with markings on cartridge cases from a known firearm to evaluate the probability that the compared patterns came from the same source, which may criminally implicate a specific person. Forensic firearms identification has been admitted in criminal trials for over a century (23, 24).

The past two decades have seen growing concern over the extent to which forensic pattern comparison methods yield accurate results. Much of this concern has arisen from the frequency with which independent evidence, such as crime scene DNA, fails to corroborate pattern testimony (25). Concern also stems from the inherently subjective nature of pattern comparison disciplines, and expanding knowledge of the sensitivities and biases characteristic of human vision, memory, and decision-making (26–32). These considerations, together with the extreme personal and societal costs of wrongful conviction, highlight an urgent need for research to evaluate whether pattern comparison methods work as advertised—a need repeatedly emphasized in recommendations from respected scientific organizations (20, 33) and much scholarly literature on this topic (e.g., ref. 34).

The practice of forensic firearms examination is based on three premises (24, 35): 1) under explosive pressure of gunpowder ignition, the machined steel surfaces of a firearm make patterned impressions in the softer metals of the cartridge case and bullet, 2) these impressions are unique to a gun and repeatable, and 3) examiners are capable of visually assessing similarities and differences between impressions on material collected as evidence and those on material from a known firearm, and making categorical judgments about common source. There is little reason to doubt the mechanical premise of pattern creation. While there are unresolved questions about the uniqueness and repeatability of patterns produced (36), most of the recent debate has focused on the third premise, which gets to the question of how well examiners perform the method of comparison (27, 37–40).

A number of experiments have been conducted to assess the validity of firearms identification. Some of these studies either do not address the accuracy question directly, being more concerned with pattern production and techniques for pattern characterization, or they are limited by design flaws and small sample sizes (41, 42). One recent study (43), which has been highly touted by firearms examiners, overcomes some of the shortcomings of prior work. This study is of considerable interest to a growing community of scientists, statisticians, and legal professionals concerned about the validity of the method, and it is rapidly gaining infamy as an antihero in our centuries-long progression toward open science. Known commonly as “Ames II,” the study was “designed and supported by the Federal Bureau of Investigation (FBI) through contract with the Ames Laboratory,” which is a research facility of the US Department of Energy (DOE) operated on the Ames, Iowa, campus of Iowa State University. The study was conducted between 2016 and 2020, and analyzed by Ames staff and university faculty. The FBI, which paid for this exercise, is an agency of the US government operating under the jurisdiction of the Department of Justice, with an annual budget in excess of $10 Billion (44). The vast majority of that budget is received by congressional appropriation from federal tax revenue.

## What Is Ames II and Why Is It Important?

Ames II is the largest study to date of the performance of forensic firearm examiners. Examiners were recruited to the study as volunteers and results were obtained from 173 participants, the majority employed by state and local crime labs with an average of 10 y of professional experience. All examiners were instructed to treat samples as they would regular casework and employ the method for comparison and classification (Identification, Elimination, Inconclusive, Unsuitable) formalized and adopted three decades ago by the Association of Firearm and Toolmark Examiners (AFTE) (35). Numerous other features of the design of Ames II, such as the independence of sample comparisons and the use of both bullet and cartridge case comparisons, positively distinguish this study from prior validation experiments.

Results from the Ames II study originally appeared in October 2020 on the DOE Office of Scientific and Technical Information (OSTI) website as Ames Laboratory‐USDOE Technical Report #ISTR‐5220 (43), which was authored by investigators from Ames Laboratory and Iowa State and “prepared for the Federal Bureau of Investigation.” This 127-page report described three phases of the study, which included measurement of accuracy (phase 1), repeatability (phase 2), and reproducibility (phase 3). Summary-level findings from phase 1 were presented at the 2021 conference of the American Association of Forensic Science (AAFS) (45). In June 2021, the technical report was submitted by federal prosecutors in a criminal case as part of an admissibility hearing challenging the scientific foundations of firearm and tool mark examination (46). Shortly thereafter, the technical report was scrubbed from the internet, and the Assistant General Counsel at the FBI admonished examiners that they could discuss the 2021 AAFS conference abstract, which contained only summary results from phase 1 of the Ames II study, but not the Technical Report (47).

In the four years since completion of the study, results from Ames II have appeared in three peer-reviewed publications. One of these (February 2022) presents the rationale for the study, the experimental design, and the complex logistics involved in carrying it out (48). This is a valuable synopsis, but it contains none of the data. The second publication (January 2023) reports on the accuracy phase of the study (49), and the third (September 2023) reports on repeatability and reproducibility (50). The latter two publications contain summary analyses and some of the raw data. (Curiously, the Ames researchers declined authorship and individual acknowledgement on the published studies. The remaining authors are employees of the FBI.)

Ames II is critically important because it ostensibly gives an indication of whether firearm identification is a valid method, and trial judges in many jurisdictions are required to keep out invalid or unproven expert evidence as part of their gatekeeping function. One factor judges consider is the “the known or potential rate of error” of the method (51). Ames II reported a false-positive (different source samples classified as the same) error rate of 0.7% for bullet comparisons and 0.9% for cartridge case comparisons, with those errors being made by a minority of the participating examiners.

## What Is Wrong with Ames II?

Ames II was explicitly designed to determine whether established methods for forensic firearms identification hold up under scrutiny, as assessed by simple quantitative measures of examiner performance. The authors of the study report that performance is highly accurate. Forensic examiners and prosecutors have brandished that statistic in battles to gain admissibility of firearms evidence in criminal trials. Scientists who have pressed for careful validation applaud the motivation behind Ames II—it was an organized response to longstanding criticism of the field—but have subjected the protocol and published results to intensive inspection. From the perspective of scientific quality, Ames II is wanting. A number of weaknesses and outright flaws have been identified, which undermine conclusions about examiner accuracy. We briefly highlight four of these problems to illustrate the range of concerns.

### “Inconclusives” Confound Error Rate Calculations.

Well over 40% of the comparisons made by Ames II examiners were reported to be inconclusive, meaning that examiners could not classify test samples as identification or elimination. We and others have argued that this approach is flawed because an examiner’s idiosyncratic placement of the upper and lower decision criteria for an inconclusive decision directly affects measures of performance accuracy (27, 38, 42, 52). Moreover, reports of low error rates based solely on identification and elimination decisions, with inconclusive judgments uncounted, are uninformative products of circular reasoning: examiners simply do well on classification problems that they find easier to judge. In other words, examiners elided over half of the presumably challenging comparisons and did well on the comparisons they elected to judge.^*^

### Sampling Biases Raise Questions about Generalizability.

Ames II relied upon volunteer examiners who, by definition, self-select for participation, which (a) makes it unsafe to assume that they are representative of the examiner community, and (b) may lead to biased outcomes (e.g., examiners who have time and interest to volunteer may be better or worse, on average, than the examiner community as a whole). A related sampling problem is attrition, which was pervasive in Ames II and may further bias the conclusions. Given these sampling problems, there is little reason to believe that results from Ames II can justify generalized claims about the performance of forensic firearms examiners (53).

### “Challenging Comparisons” Failed to Challenge.

It has recently come to light that many nonmatching comparisons in Ames II employed test samples that differed in class characteristics,^†^ contrary to the initial claims of the investigators (54). It is recognized to be “very easy” to determine that cartridge cases with different class characteristics were not fired by the same gun (hence a very low probability of a false positive result). Thus, despite assertions that the study was designed to present “challenging comparisons,” estimates of performance accuracy were based on a significant number of tests that were easily classifiable as eliminations. This fact weakens assertions about examiner accuracy, and the very existence of it undermines the credibility of the investigators.

### Repeatability and Reproducibility Are “Rather Weak”.

Concerns have been raised about reported estimates of performance repeatability and reproducibility. Repeatability is a measure of intraexaminer consistency, assessed as the probability that an examiner comes to the same conclusion upon repeated testing with the same samples. Reproducibility is a measure of interexaminer consistency, assessed as the probability that different examiners come to the same conclusion when confronted with the same samples. Despite claims that consistency of intra- and interexaminer decisions is high, evidence indicates that Ames II investigators came to that conclusion erroneously (55). In fact, it appears that both same and different examiners manifest a poor ability to make the same classification decision upon repeated receipt of the same input, which casts significant doubt on reported estimates of performance and, most importantly, on the general wisdom and fairness of admitting firearm identification testimony in a court of law.

All of these weaknesses pale by comparison to what has emerged as an egregious failure of this scientific undertaking, namely the explicitly stated opposition to unfettered access to all of the raw individual subject data from a publicly funded research project, the results of which have been used in court to secure criminal convictions that carry significant terms of imprisonment (56). To be clear, the data in question raise no national security concerns; these are not scientific discoveries that, in the wrong hands, could facilitate heinous crimes or cause loss or injury to individuals, corporations, or national interests. Nor are there any concerns about financial loss from disclosure of proprietary information. On the contrary, complete datasets from forensic validation studies simply provide a means for the larger scientific community and other interested members of the public to evaluate claims of accuracy—claims that can potentially sway decisions about the admissibility of firearms evidence in a criminal proceeding.

Finally, we note that some of the problems hinted at above—sampling biases, misuse of statistics, weak experimental design—are not unique to Ames II and we do not single the study out for that reason. On the contrary, we draw attention to it because failure to share the complete dataset in this case precludes the full accountability that a democratic society demands for high-stakes decisions.

## What Specific Information Is Needed and Why?

The Ames II report contains various rates of error, repeatability, and reproducibility. These rates are summaries of the raw data. That is, individual responses were aggregated and subjected to various mathematical calculations. The results of these calculations were then reported. The concerns highlighted here (and others previously noted) may be resolvable, and the broader substance, significance, and probative value of the study made more apparent by affording access to individual subject responses to all stimuli in all conditions that were tested. There are several specific reasons why this is important, which we highlight here.

The most straightforward reason why access to these raw data is important is the need for independent confirmation of the accuracy of published summary values that were calculated from individual examiner data. Errors unwittingly happen when working with large datasets. Indeed, as noted above, a careful review of published results on Ames II examiner accuracy (50) revealed that many test samples were misclassified: they were said to be “challenging” same-class comparisons, but were in fact “very easy” different-class comparisons (54). The authors ultimately acknowledged that different-class comparisons may have been included in the study (57). We appreciate that errors can cut both ways, but the extent to which they exist can only be fully and independently assessed if open access is provided to the complete dataset.

Concerns about sampling bias may also be addressed, or at least better understood, by providing access to all individual examiner data. Scientific studies commonly seek samples that are representative of populations of interest, such that results obtained from a study sample can be safely generalized to the entire population (58). Whether this can be accomplished in practice depends on many different factors. One factor is attrition, which was alarmingly high in Ames II:

“A total of 173 examiners working in 41 states returned evaluations and were active in some part of the study. At the conclusion of the study, only 79 participants had finished all six mailings of test-packet analyses.”^‡^

The chief concern is whether examiners who complete the study differ in material ways from examiners who do not finish, resulting in an unrepresentative sample. As noted by two statisticians in a recent peer-reviewed article (42), “The key consideration is whether the act of responding is related to what is being measured.” For example, did the most proficient examiners continue while less proficient examiners dropped out? One can easily see how this would distort the reported error rates: if the most proficient examiners completed the study, then overall error rates would be lower than if the less proficient examiners completed the study and their results were included. This is not just a theoretical possibility: Law and Morris (59) reported results of a firearms study in which one examiner committed five false positive errors and then withdrew from the study. The authors note that “due to this participant's decision to withdraw, [their data] will not be included in the remainder of the analysis.” In contrast to this unfortunate practice, access to the Ames II raw data would enable independent analysis of the effects of attrition on the professional experience and performance of those who completed the study versus those who did not, and an inquiry into the particular conditions and test samples that were not completed by those who dropped out.

Access to the complete individual examiner dataset would also provide insights into the worrisome questions of consistency of classification decisions. For example, do the more experienced examiners exhibit higher self-agreement when they reexamine the same bullets or cartridge cases? Does the type of laboratory (e.g., state vs. federal) affect rates of repeatability and reproducibility? What are the effects of attrition on repeatability and reproducibility of classification decisions? Access to raw data also bears on interpretation of inconclusives. As treated in Ames II, these classification decisions can artifactually reduce computed error rates. There is no simple or agreed-upon solution to this problem, but the data may provide valuable insights. For example, is there an interaction between inconclusive decisions and consistency of reporting at the individual examiner level?

Finally, and perhaps most importantly, access to the raw data can allow investigators to determine characteristics of the extremely “poor performers” in the Ames II study. The authors noted that a “relatively small subset” of examiners (20%) committed false positive or negative errors in the study (43). However, the number of errors committed by these examiners is shocking. For example, these examiners committed a false positive error in 21% of the bullet comparisons and 17% of the cartridge case comparisons they conducted in the study (60). Ames II reported that examiner experience (measured in years) was unrelated to accuracy but failed to examine whether any other factors indicated poor performance. Being able to identify poor performers and keep them from testifying is critical.

## Priority of Publication

A common argument from an earlier time in which data-sharing mandates scarcely existed was that scientific investigators should be permitted time to obtain priority of publication before releasing the data upon which a publication is based (61). In response to a motion to compel the release of the full Ames II dataset,^§^ the government made precisely this argument:

“Researchers who publish in the prestigious journals that declarants reference would likely be horrified to be forced to share the raw data of their studies before they publish their own results and analysis. This would have an exquisite chilling effect on publishing first account results and undermines the integrity of the scientific process.” (62)

The argument is not entirely without merit, but when weighing professional rewards against the value of open science for informing fateful decisions, funding agencies and regulatory authorities today come down on the side of rapid data sharing. For example, as discussed below, the NIH and the Food and Drug Administration (FDA) now require that all data from clinical trials—validation studies for the effectiveness of drugs—be publicly posted within 1 y following completion of data collection.

The government’s response to the motion to compel also betrays a misunderstanding of the functional utility of timely data sharing:

“It would also undermine the integrity of the study by allowing outsiders with unknown motives to rework the data to their own ends before the scientific process has completed a peer-review endorsed result. As a matter of scientific integrity, it is unreasonable to disclose the underlying data before that scientific process is complete.” (62)

To be clear, it is in the service of “scientific integrity” that open science exists. “Open science is the idea that scientific knowledge of all kinds should be openly shared as early as is practical in the discovery process.” (63) Doing so enables other parties to evaluate the quality, credibility, and significance of data before it is used for decisions and actions that may have profound personal or societal consequences. In a democratic society, open science affords public accountability at the time that matters most.

The two published reports that contain data from the Ames II study appeared in 2023 (49, 50). It would be hard to argue that the investigators did not have sufficient time to produce these reports sooner, particularly since the now-ghosted technical report from 2020 included analyses of repeatability and reproducibility, as well as accuracy. By today’s open-source standards, however, peer-reviewed summary reports of digested data are insufficient. It is the complete raw dataset that affords accountability.

## Anonymity of Participants

A longstanding concern in experiments with human subjects is that personal details or an identified subject’s task performance could be included in data that are publicly shared. In response to the motion to compel the release of the raw data from Ames II (64), the government echoed this concern: “the ‘raw data,’ if released prematurely and prior to the completion of the peer review process, would reveal the identities of the hundreds of participants of this study” (62). The government furthermore maintained that “Revealing the raw data at this juncture would be a breach of ethical duties and would create a chilling effect in future research, as examiners would be less likely to volunteer if they believed their identities would not be protected.”

This rationale for withholding raw data is a red herring. By federal regulation, Institutional Review Boards (IRBs) provide oversight and protection of human subjects. In particular, the Department of Health and Human Services (HHS) “Common Rule” contains provisions to restrict the release of personal identifiers. Moreover, the identities of examiners who participated in Ames II are irrelevant to the scientific concerns that have been raised and to an independent audit of individual examiner classification decisions and summary calculations. It has long been expected practice to anonymize raw data collected in experiments designed to validate the effectiveness of drugs and medical devices. There are standard procedures for doing so, which apply equally well to forensic validation studies, such as Ames II.

## A New Open Science Imperative: Due Process

As we highlighted at the outset, historians and social scientists have identified five “schools of thought” that constitute the canon of open science: public engagement, democratic right of access, efficiency of knowledge gain, shared technology, and better assessment of impact (5). The problem posed by the refusal to share data from a large publicly funded study of the validity of forensic techniques identifies a sixth school of thought for inclusion in the open science canon—one that is born from the very foundation of our criminal justice system: Due Process. The core principle of due process under the law, enshrined in the US Constitution, is fairness. This fairness doctrine takes several forms, including preservation of rights and immunities afforded to citizens of the Unites States, by adoption and enforcement of processes “to minimize substantively unfair or mistaken deprivations” (65), and equal protection of the law for all people within the jurisdiction of the United States.

In 1963, the US Supreme Court ruled in *Brady v. Maryland* (66) that one element essential to due process under the law is “discovery,” which sensibly maintains that fairness in litigation depends on all parties having equal access to information that may influence the outcome. *Brady* established a right to discovery, implemented by the “Brady Rule.” Initially, Brady disclosures focused on information held by the prosecution that might be exculpatory for the defense. In later rulings (67, 68), the Court adopted an explicit materiality standard for determining whether a piece of information should have been shared: “the evidence is material only if there is a reasonable probability that, had the evidence been disclosed to the defense, the result of the proceeding would have been different.” It is the defendant’s burden to prove materiality, but meeting this standard constitutes a “Brady violation,” which can lead to a mistrial.

Although evidence used in criminal litigation comes in many forms, when that evidence is scientific, the due process Brady Rule is de facto an open science mandate, which places the parties on equal informational footing and positions the trier of fact to make decisions that fairly benefit individuals and societies.

## Ames II and the Right to Discovery

To their credit, the authors of the Ames II study finally released *some* of the larger dataset in their 2023 articles. We remain hopeful that the complete body of individual subject data will be made public, but the timing is particularly problematic because the real-world consequences of the failure to share data now stretch far beyond the ivory tower. With growing recognition by a community of scientists and statisticians that forensic pattern comparison methods have weak scientific foundations, admissibility hearings have become highly contentious battles between forensic examiners and bench scientists (40). But the cards are stacked: For some time now, government prosecutors have used summary conclusions from the Ames II study to argue for the admissibility of firearms evidence in criminal trials, while much of the underlying data for those conclusions are held in check by the law enforcement arm of the Department of Justice. More generally, as noted in a recent scholarly report on open forensic science efforts:

“There is well-established imbalance in the state’s ability to develop a forensic scientific case against an accused and the accused’s ability to assess that case and amass his or her own evidence. This inequity is heightened when the foundational science behind the state’s case was conducted opaquely and published in paywalled journals.” (21)

To illustrate the tragic consequences of this state of affairs, consider the use of firearms evidence in a recent trial in federal court (56). The defense twice moved to compel the release of raw data from Ames II for review, which would have enabled experts for the defense to detect the fact that, contrary to the Ames II report, estimates of performance accuracy were based on a significant number of tests that were easily classifiable (54). The judge denied the motion, stating that the need to check the data for unwitting errors is “speculative” and concluding that the raw data could not be used “to determine bias in participant drop out rates” (69). The judge instead credited the testimony of Erich Smith, an FBI firearm examiner who was a coauthor of the 2021 Ames II AAFS presentation and the three subsequent peer-reviewed publications:

“Smith testified that ‘a fair conclusion’ is ‘that no matter the study design, no matter what the study conditions are, the false-positive rate is consistently around 1 percent or less.’” (56)

The judge concluded that “the error rate for toolmark analysis weighs strongly in the Government's favor” and admitted the firearm evidence without any restrictions. Both defendants were convicted and sentenced to mandatory life imprisonment without parole (70).

As summarized above, even the limited information currently available about Ames II points to statistical flaws (27, 38, 42, 53, 55), sampling biases (42, 53), and misrepresentation (54, 57, 60). All of which would seem to meet the materiality standard for discovery (67, 68). It naturally follows that if the prosecution has unilateral access to the unpublished individual subject dataset from Ames II—as was true for the FBI examiner who testified in the federal firearms case cited above—the prosecution is at risk of a Brady violation of the defendant’s Constitutional right to due process.

## The Clinical Trial: A Model for Large-Scale Validation of Scientific Inventions

We advocate here for public release of all data from a large-scale scientific study designed to validate the effectiveness of an invented instrument that has profound societal implications. This is, of course, not unprecedented. Over the past century, our society has witnessed the evolution of a sophisticated system for scientific validation and reporting: The Clinical Trial, which provides an empirical foundation for medicine, one of the world’s most successful applied sciences. There are large matters of safety and efficacy to be proved, and issues of trust and responsibility that must be navigated to gain public support. Data sharing has been the primary instrument of change in the way that clinical trials are performed, interpreted, and used to gain drug approval, and this practice stands as an exemplar of modern open science. The details closely parallel the forensic problem and offer a clear-eyed path forward.

The therapeutic effectiveness of clinical tools in medicine has long been evaluated by physician reports published as case studies in scholarly journals. In 1938, Congress passed the Food, Drug, and Cosmetic Act, which was more concerned with safety than efficacy, and imposed no requirement for FDA approval to move forward with marketing. The mid-20th century saw the emergence of more rigorous approaches in which novel statistical methods were introduced to assess the impact of medical treatments on disease progression. In 1944, the Council on Pharmacy and Chemistry of the American Medical Association (AMA) published a landmark report entitled “Laboratory and Clinical Appraisal of New Drugs” (72), aimed at promoting sensible standards for scientific evaluation. The opening paragraph of this report offers a broad but illuminating definition of a clinical trial:

“A new drug should pass through several phases of investigation before it is declared suitable for distribution in commerce. It should be studied in the laboratory and in the clinic, the details of the study depending on the nature of the ingredients and the intended uses, but *all investigations should follow a general plan which will permit a thorough understanding of the usefulness and toxic properties of the drug*.” (emphasis added)

The AMA report went on to note that the FDA at that time was primarily concerned with drug safety (“toxic properties”) but had no authority to impose requirements for the validity of the claimed clinical effects (“usefulness”) of a drug. Nonetheless, in recognition of the critical importance of proving validity, the AMA stressed the need for carefully planned and well-controlled experimental design and analyses:

“The Council on Pharmacy and Chemistry is concerned not only with the evidence of safety but also the evidence adduced to support the claims made for new drugs. Too frequently this evidence is found inadequate and the sponsors of new preparations, if they wish to provide the missing data, may find it necessary to repeat some of the more time-consuming and expensive procedures and at other times find it advantageous to proceed along entirely new lines of thought.”

“it is necessary to develop methods of appraising the therapeutic usefulness and potential harmfulness of new drugs and to organize these methods into a logical system which, if followed, will give reasonable assurance that the new preparation will not be offered to the medical profession or to the public before the extent of its usefulness or the potentialities for harm are understood.”

The AMA argued, furthermore, for transparency and openness when documenting methods, experimental conditions, and results of validity testing:

“it is an advantage for physicians and allied scientists to know by what standards a new drug has been evaluated. When the physician is urged to use this agent, he should have available such evidence as will satisfy his questions concerning safety and efficacy.”

“Above all, it should be realized that summaries of case histories *unless accompanied by the full report* from which the summaries were derived are of little significance.” (emphasis added)

The AMA’s emphasis on experimental design and transparency was transformative, but it was not until after the Second World War and expansion of medical research funding by the NIH that randomized controlled trials (RCTs) were adopted on a large scale for clinical validation. Subsequently, and largely in response to the thalidomide tragedy of the 1960s—and to address a longstanding commercial affinity and American gullibility for snake-oil therapeutics (73)—Congress enacted in 1962 the “Drug Efficacy Amendment” to the Food, Drug, and Cosmetic Act, a milestone of consumer protection legislation that set forth new policy for FDA approval of clinical tools (74). That policy dictated a “proof of efficacy” requirement, which heralded the onset of modern standards and afforded regulatory teeth to ensure that drugs and medical devices actually do what they are expected to do.

In recognition of the utility of clinical trial methods and data for informing decisions about the use of a drug, the 1944 AMA report highlighted the need for sharing “the full report.” This open science sentiment has grown significantly in the intervening decades, with the understanding that data sharing enables independent verification or refutation of efficacy and safety by other investigators, affords broader and more coherent development of policies for use of a drug, improves tools for measurement, analysis, and modeling, and elicits public confidence in discoveries. In 1985, the Committee on National Statistics of the National Research Council (NRC) published a report calling for data sharing in both publicly and privately funded science (75). The report considered both the costs (e.g., technical and administrative burdens, loss of revenue from patents and licensing, forfeiture of rights to priority of publication), and the “manifestly clear and widely accepted” benefits of data sharing, and came down strongly on the side of open science, with recommendations for means to promote that practice and lessen the onus on scientists.

## Uniform Policies for Data Sharing with Enforcement

The NRC’s watershed 1985 report led, over the next four decades, to broad open science policy declarations by critical stakeholders (professional organizations, funding agencies, vehicles for science communication). The National Academies of Science, Engineering, and Medicine returned to this topic in 2018, noting that “the research enterprise has already made significant progress toward open science, and is realizing a number of benefits, with the expectation that these will expand in the future” (3).

The sentiments at the heart of these policies and progress are widely held, but scientific publication has long been plagued by selective reporting of results and resource limitations (76, 77). In part for this reason, regulatory and funding agencies and journals have moved to establish firm and explicit requirements for data sharing. These requirements—e.g., the sharing requisite imposed on authors by the *PNAS* (78) and, more recently, by the *Nature* (79), *PLoS* (80), and *Science* (81) families of journals (e.g., *Science* magazine)—are now directed at all forms of scientific research. Their application to clinical trials has, nonetheless, taken the lead in establishing and enforcing rules because of legitimate societal concerns about the efficacy and safety of drugs and medical devices (4).

In 2007, Congress set the stage for extensive data sharing through the enactment of the FDA Amendments Act (FDAAA) (8), which requires registration and approval of a planned clinical trial, and posting of methods, conditions, and results upon completion. The FDAAA was clarified and complemented in 2016 by a regulation issued by HHS, known as the “Final Rule for Clinical Trials Registration and Results Information Submission” (82). The Final Rule imposes additional sharing requirements, including mandates for publicly posting all data within one year of study completion, and not limited to studies for which drugs are actually approved and licensed. The NIH followed suit by adopting a “Policy on Dissemination of NIH-Funded Clinical Trial Information” (83). Effective January 2023, NIH adopted a more general “Policy for Data Management and Sharing (DMS Policy) to promote the management and sharing of scientific data generated from all NIH-funded or conducted research” (84). This policy states that “shared scientific data should be made accessible as soon as possible, and no later than the time of an associated publication, or the end of the award/support period, whichever comes first.” The NSF maintains a similar policy (85).

Lest one choose to shirk this responsibility to open science, the FDA warns:

“These statutory and regulatory requirements are intended to provide greater transparency regarding clinical trials, ultimately allowing the broader scientific community to build on the information submitted. The submission to and posting of clinical trial information on ClinicalTrials.gov honors volunteers who participate in research to advance medical science and enhances public trust by creating a transparent and robust public record of clinical trials and information about their results. *When these legal requirements are not met, the FDA has the authority to take enforcement action.*” (86) (emphasis added)

Indeed, it has done so: The FDA issued its first Notice of Noncompliance to Acceleron Pharma in 2021 for failure to submit “required clinical trial results information in the manner and format specified” (87), and continues to track compliance with its data sharing mandate, with the explicit threat of civil penalty under federal law.

## Recommendation: The Forensic Trial

In this perspective, we make a broad call for open science in forensic validation studies. We do so in the context of the urgent need for data sharing from a government-funded study that is now influencing legal policy and practice. We also offer specific recommendations for how forensic disciplines can use science to gain public confidence in practices that play a critical role in law enforcement. A forensic validation study is, by analogy to the clinical trial, a “Forensic Trial” intended to assess the safety (false alarm rate) and efficacy (hit rate) of an invention designed to treat a societal ill. The regularized structure of clinical trials for drug development stands as a model for the conduct of validation studies generally—one that forensic disciplines would do well to emulate.

To convey how this could work and why it is so important, we turn to a 2015 consensus report from the Institute of Medicine that focused on sharing of data from clinical trials:

“Responsible sharing of clinical trial data is in the public interest. It maximizes the contributions made by clinical trial participants to scientific knowledge that benefits future patients and society as a whole. Results from many clinical trials are not published in peer-reviewed journals in a timely manner. Even when findings are published, large amounts of data remain unanalyzed. Data sharing makes data from clinical trials available to other investigators for secondary uses, which include carrying out additional analyses, analyzing unpublished data, reproducing published findings, and conducting exploratory analyses to generate new research hypotheses. In several highly publicized cases, independent investigators who have reanalyzed the data underlying published results of clinical trials have challenged the published results as invalid or incomplete. These allegations have sparked debates, additional analyses, and new clinical trials. Further, they have caused regulators to limit marketing of the products or led sponsors to withdraw them. This back-and-forth discussion, while complex and perhaps confusing to the public, is how scientific knowledge progresses, and it has resulted in a broader evidence base for regulatory and clinical decisions.” (4)

Substitute “forensic” for “clinical”—as in, “forensic trials” and “forensic decisions”—and the arguments made by this committee apply with the same reason and force to the empirical validation of forensic pattern comparison disciplines. This is, indeed, “how scientific knowledge progresses,” and it is surely the way to achieve a “broader evidence base for regulatory and [forensic] decisions.” Following this model, we recommend the following:

(1) The design of all forensic trials must be registered with funding agencies or published as peer-reviewed “registered reports” before commencing (88–90). This will afford a valuable opportunity for design review by the scientific community, along with subsequent ability to detect deviations from the prespecified design and data analytic plan (91), and
(2) All forensic trial data—summary-level data, raw data, and the analyzable dataset (e.g., deidentified individual participant data from studies with human subjects), and metadata, or “data about the data” (e.g., protocols, statistical analysis plans, and analytic code) (4)—must be posted to a publicly accessible site within one year of completion of data acquisition.

The existence of a large federal agency—the FDA—makes these practices feasible and enforceable in the case of clinical trials. A recommendation for an analogous oversight and regulatory agency for forensics—a National Institute of Forensic Science—was made by the 2009 NAS consensus report committee on forensic science (20). There are complex political and budgetary issues raised by the prospect of this federal agency, and it has not come to pass. There are, nonetheless, plentiful opportunities for professional organizations, funding agencies, and journals to leverage transparent registration and free reporting of data from forensic trials.

Finally, we note that there already exists a regulatory and enforcement mechanism that can be applied to the forensic data sharing problem: the gatekeeping trial judge. Empowered by the 1993 Supreme Court ruling in *Daubert v. Merrell Dow Pharmaceuticals, Inc.* (51) trial judges are responsible for applying quality standards for the admission of scientific evidence to a court of law, the primary goal of which is to ensure that the information is a trustworthy basis for decision. We thus make an additional recommendation:

(3) Trial judges should consider refusal to share data from forensic trials to be highly suspect and outside the norms of science, which (per *Daubert*) disqualifies relevant scientific testimony as a trustworthy basis for legal decisions.

This third recommendation is not unprecedented. Judges in other domains have excluded scientific evidence when experts refuse to turn over the raw data (92).

## Unintended Consequences of Open Science in Forensics?

Open science is an ideal but, as Bacon himself noted, there may be unintended consequences of knowledge gain. Suppose, for example, that the complete dataset in Ames II reveals that forensic firearms identification has limited accuracy. This might shift the decision criterion for admissibility of firearms evidence, such that fewer innocent suspects are prosecuted and convicted. At the same time, a larger fraction of guilty suspects will go free, which may facilitate greater gun violence. This is a signal detection problem, at heart, for which there is no perfect solution. Under conditions of uncertainty, there will always be a probabilistic tradeoff between wrongful conviction and failure to convict the guilty. Science has little to say about the choice that a society makes along this continuum. But knowledge is power, and these societal decisions must always be made in the context of free and equitable access to the fruits of scientific research. Indeed, true justice—however a society defines it—demands open science.

Open science offers a path to a more just future, but it may also force us to reckon with the past. Were the Ames II dataset to reveal limited accuracy, we would face the demoralizing prospect that some prior convictions were based on flawed evidence. Postconviction DNA analyses have already begun to address this issue through the courts (25), but a true scientific understanding of the validity of forensic pattern comparison tools may place our criminal system at a watershed moment for reform.

## Data Availability

There are no data underlying this work.

## Footnotes

^*^Michael Rosenblum, a statistician at Johns Hopkins University “compares the situation to one in which students taking the SAT were allowed to skip any question they couldn’t answer. ‘It’s not a real test; everyone will get a perfect score’” (71).

^†^Class characteristics are attributes common to a subgroup or class of items, such as impressions made by the same model of gun.

^‡^256 participants initially enrolled. Only 173 provided responses, 79 of whom completed the study. Thus 69% of enrollees failed to complete the study.

^§^Motion made by defense in firearms admissibility hearing for *United States v Ronald Clayton Rhodes and Lorenzo Laron Jones*. (Case no. 3:19-CR-00333-4-MO) Dist. of Oregon (56). Scurich served as expert witness.
